# TAKING A BREAK: DAILY RESPITE EFFECTS OF ADULT DAY SERVICE AS ACTUAL TIME AWAY FROM CAREGIVING

**DOI:** 10.1093/geroni/igz038.2209

**Published:** 2019-11-08

**Authors:** Molly J Wylie, Kyungmin Kim, Yin Liu, Steven H Zarit

**Affiliations:** 1 University of Massachusetts Boston, Boston, Massachusetts, United States; 2 Utah State University, Logan, Utah, United States; 3 Pennsylvania State University, University Park, Pennsylvania, United States

## Abstract

Adult day service (ADS) can provide emotional and physical relief for caregivers of persons with dementia (PWD). Studies have examined differences between caregivers of service users and non-users; less known, however, is how actual hours away from caregiving responsibilities through using ADS impact caregivers’ daily outcomes. Using daily diary data from 173 family caregivers whose relatives are using ADS (day N = 1,359), this study investigated within-person differences in respite hours across 8 consecutive days and how daily respite hours are associated with daily well-being (i.e., mood and health symptoms). On average, caregivers reported 7.12 respite hours on ADS days and 1.74 respite hours on non-ADS days. Multilevel models revealed that having more respite hours is associated with better positive mood, but not with negative mood and health symptoms – after controlling for ADS use. Further, when caregivers perceived more break time from caregiving responsibilities, they showed better positive mood.

